# In patients with localized bronchiectasis, does thoracoscopic lung resection result in improved clinical outcomes compared to open surgery?

**DOI:** 10.1093/icvts/ivab329

**Published:** 2021-11-24

**Authors:** Yasser Ali Kamal, Hussein Elkhayat

**Affiliations:** 1 Department of Cardio-thoracic Surgery, Minia University, El-Minya, Egypt; 2 Department of Cardio-thoracic Surgery, Assiut University, Assiut, Egypt

**Keywords:** Bronchiectasis, Video-assisted thoracoscopic surgery, Thoracotomy, Lung resection, Postoperative outcome

## Abstract

A best evidence topic was constructed according to a structured protocol. The question addressed was whether video-assisted thoracoscopic surgery (VATS), compared to open lung resection, resulted in improved postoperative clinical outcomes, in patients with localized bronchiectasis indicated for surgery. A total of 1352 papers were found using the reported search, of which 5 papers represented the best evidence to answer the clinical question. The authors, journal, date and country of publication, patient group studied, study type, relevant outcomes and results of these papers are tabulated. All of the identified studies were retrospective. The conversion rate from VATS to open procedure ranged from 0% to 15.3%. In comparison to thoracotomy, VATS resulted in a significant reduction of postoperative blood loss (1 study), length of hospital stay (2 studies), postoperative complication rate (2 studies), pain scales (2 studies) and chest tube duration (1 study). However, there was a significant increase in operative time (1 study) and whole cost (2 studies). There was no significant difference in the clinical recovery, which was defined by the disappearance or improvement of preoperative symptoms during the follow-up period (3 studies). We conclude that although there is limited high-level evidence, retrospective studies have suggested that VATS could significantly reduce complications rate, postoperative blood loss, pain scales, length of hospital stay and chest tube duration compared to open lung resection in selected patients with localized bronchiectasis.

## INTRODUCTION

A best evidence topic was constructed according to a structured protocol. This is fully described in the interactive cardiovascular and thoracic surgery (ICVTS) [1]. 

## THREE-PART QUESTION

In [patients with localized bronchiectasis indicated for surgery], does [video-assisted thoracoscopic surgery (VATS)], compared to [open lung resection], lead to improved postoperative [clinical outcomes]?

## CLINICAL SCENARIO

A 24-year-old female patient was admitted to the cardiothoracic surgery department to manage a bronchiectasis lesion at the left lower lobe with minimal scarring and adhesions on high resolution computed tomography (HRCT). A surgical colleague suggested thoracoscopic lobectomy (TL) in such cases. We had no idea about the evidence and decided to investigate further.

## SEARCH STRATEGY

A comprehensive literature search was conducted using the PubMed interface from 1935 to June 2021. The employed search strategy was: [(bronchiectasis) OR (infectious lung disease)] AND [(thoracoscopic surgery) OR (VATS) OR (lung resection) OR (lobectomy)]. The eligible papers were randomized controlled trials, prospective studies, retrospective studies or case–control studies in the English language. Case reports, studies on diffuse bronchiectasis, studies that did not include the outcome of interest (complications rate or clinical recovery defined by symptomatic relief) and studies that did not compare VATS to open procedure were excluded.

## SEARCH OUTCOME

A total of 1352 papers were identified using the reported search. After screening of the relevant papers and the reference lists, 5 papers were identified that provided the best evidence to answer the question. All these papers are presented in Table 1.

**Table 1:** Best evidence papers

Author, date, journal and country Study type (level of evidence)	Patient group	Outcomes	Key results (VATS versus thoracotomy)	Comments
Weber *et al*. (2001), Eur J Cardiothorac Surg, Switzerland [2] Retrospective study (level III evidence)	117 patients (benign diseases; 54 bronchiectasis) VATS: 64 Thoracotomy: 53 Exclusions for VATS: Inadequate cardio-pulmonary conditions, non-elective operation, severe parenchymal and (or) pleural scarring on computed tomography (CT)	Operative time (h)	2 (0.5–6) vs 2.5 (1–4), *P* > 0.05	The inclusion of other bengin diseases limits the study
		Blood loss (ml)	0 (0–2000) vs 300 (0–6000), *P* > 0.05	
		Length of hospital stay (days)	8.5 (4–41) vs 10 (5–52), *P* > 0.05	
		Drainage time (days)	5 (1–32) vs 6 (3–21), *P* > 0.05	
		Complications rate (%)	18.7 vs 43.4 (*P* < 0.05)	
Zhang *et al*. (2011), Ann Thorac Surg, China [3] Retrospective study (level III evidence)	104 patients (propensity scores matched) VATS: 52 Thoracotomy: 52 Exclusions for VATS: Absence of aesthetic demands, purulent sputum lasted >5 years, severe parenchymal and (or) pleural scarring on CT	Operative time (min)	143.5 ± 82 vs 156 ± 95 (*P* = 0.056)	-
		Blood loss (ml)	126 ± 70 vs 130 ± 54 (*P* = 0.06)	
		Length of hospital stay (days)	11 ± 6.5 vs 14 ± 12 (*P* = 0.04)	
		Cost (10^5^ RMB)	5.4 ± 1.2 vs 4.6 ± 0.7 (*P* < 0.001)	
		Pain scales at POD 14 (mean)	1 vs 4 (*P* < 0.05)	
		Complications rate (%)	15.3 vs 26.9 (*P* = 0.03)	
		Clinical recovery: complete absence of preoperative symptoms (%)	94.2 vs 88.4 (*P* = 0.57)	
Zhou *et al.* (2013), Chin Med J (Engl), China [4] Retrospective study (level III evidence)	56 patients cVATS: 35 Thoracotomy: 21 Exclusions for VATS: Severe scarring or adhesions on CT	Operative time (min)	168.7 ± 55.9 vs 204.3 ± 89.7 (*P* = 0.072)	The 2 groups of patients may not have been well balanced
		Blood loss (ml)	210.1 ± 213.1 vs 464.3 ± 536.5 (*P* = 0.01)	
		Postoperative stay (days)	8.5 ± 3.5 vs 9.2 ± 4 (*P* = 0.49)	
		Cost (RMB)	43 430 ± 5768 vs 31 129 ± 6072 (*P* < 0.0001)	
		Complications rate (%)	17.1 vs 33.3 (*P* = 0.20)	
		Clinical recovery: Asymptomatic plus significant symptom improvement (%)	94.3 vs 90.5 (*P* = 1)	
Hao *et al.* (2019), Chin Med J (Engl), China [5] Retrospective study (level III evidence)	99 patients VATS: 49 Thoracotomy: 50	Operative time (min)	154.08 ± 53.38 vs 129.23 ± 42.94 (*P* = 0.01)	The patients in the VATS group were older
		Blood loss (ml)	563.47 ± 625.49 vs 499.17 ± 708.03 (*P* = 0.62)	
		Length of hospital stay (days)	8.17 ± 3.45 vs 9.78 ± 4.25 (*P* = 0.03)	
		Chest tube duration (days)	5.90 ± 3.98 vs 8.34 ± 4.72 (*P* = 0.005)	
		Complications rate (%)	16.3% vs 18% (*P* = 0.671)	
		Clinical recovery: symptoms disappeared (%)	71.4 vs 76 (*P* = 0.694)	
Ceylan *et al.* (2021), Indian J Surg, Turkey [6] Retrospective study (level III evidence)	50 patients VATS: 20 Thoracotomy: 30	Operative time (min)	226.9 ± 93.8 vs 245.9 ± 90.5 (*P* = 0.487)	The selection criteria for VATS were not clear
		Length of hospital stay (days)	6.86 ± 4.24 vs 9.24 ± 7.12 (*P* = 0.212)	
		Mean drainage time (days)	6.33 ± 4.14 vs 8.44 ± 7.06 (*P* = 0.254)	
		Pain scales at POD 5 (mean)	1.5 vs 2.5 (*P* < 0.05)	
		Complications rate (%)	38.9 vs 37.5 (*P* = 1)	

cVATS: complete VATS; POD: postoperative day; RMB: Renminbi; VATS: video-assisted thoracoscopic surgery.

## RESULTS

The retrieved 5 papers compared VATS to open lung resection in selected patients with localized bronchiectasis. All of the studies were retrospective in nature. The absence of the randomized trials increases the bias in the procedure selection. Moreover, the selection for VATS was based mainly on the surgeon's judgement and the severity of scarring or adhesions on HRCT. This means that the VATS cases were likely to be selected to be more accessible and therefore impact the interpretation of outcomes.

Weber *et al.* [2] retrospectively analysed the results of 64 patients who underwent TL and compared this group with 53 patients who underwent open lobectomy (OL) for benign lung disease including bronchiectasis in the most of patients (36 TL; 18 OL). The preoperative work-up included chest X-ray and CT in all of the patients. The pulmonary function was determined in all elective cases, and a perfusion lung scan was added in cases of respiratory limitation. Patients were selected for VATS based on stable cardiopulmonary conditions and elective priority of the procedure. The radiological signs of severe scarring and adhesions on CT indicated the need for thoracotomy.

The conversion rate from VATS to thoracotomy was 15.3% due to severe adhesions. The differences in operation time, blood loss, drainage time and hospital stay between the 2 groups did not reach statistical significance. The nonfatal complications occurred in 18.7% of the TL group and 43.4% of the OL group. The complications include prolonged parenchymal fistulas (9.3% after TL and 9.4% after OL), haemothorax (1.5% after TL and 13.2% after OL), reoperation (4.7% after TL and 13.2% after OL), postoperative pneumonia (4.7% after TL and 15% after OL) and postoperative cardiac arrhythmias (3.1% after TL and 5.6% after OL). There was no mortality in the TL group, and a trend towards less blood loss, shorter drainage time and hospital stay was noted. More than 2/3 of bronchiectasis patients were successfully treated with VATS. The subgroup of patients operated on for bronchiectasis and undergoing middle lobe resection had a conversion rate of 6.25%, an overall complication rate of 10% and a significantly shorter operation time. The authors concluded that VATS is safe in chronic inflammatory diseases, especially bronchiectasis, with a low conversion rate, rapid postoperative recovery and favourable cosmetic results.

Zhang *et al.* [3] selected 52 from 279 patients who underwent thoracotomy and compared them with 52 patients who underwent VATS lobectomy during the same period. Although the 2 groups of patients were propensity matched, the preoperative FEV1 was almost statistically better in the VATS group (*P* = 0.055). The main selection criteria for VATS include young age with aesthetic demands, absence of severe parenchymal and (or) pleural scarring with no calcified lymph nodes near pulmonary arteries and veins on HRCT, and <5 years duration of purulent sputum. Patients with severe parenchymal and (or) pleural scarring often underwent the open procedure. VATS was converted to thoracotomy in 7 patients (13%) due to bleeding, fused fissure or hilar lymphadenopathy. The measurement of postoperative pain by an 11-point pain scale (0 = no pain, 10 = maximal imaginable pain) during the first 14 postoperative days revealed that VATS patients had significantly less postoperative pain than those in the thoracotomy group. Moreover, patients with VATS had a shorter length of stay in hospital (11 ± 6.5 vs 14 ± 12 days, *P* = 0.045), and fewer complications (8 vs 14, *P* = 0.039) than those with thoracotomy. The complications after VATS include empyema cured by chest tube insertion and pleural lavage (*n* = 1), postoperative lung torsion needed completion pneumonectomy (*n* = 1) and other minor complications (*n* = 6), which were cured by conservative therapy. The average follow-up period was 42.6 months (range: 12–64 months). In comparison with thoracotomy during the follow-up period, VATS resulted in a higher proportion of full recovery (complete absence of preoperative symptoms that led to surgery) but with no statistical difference (94% vs 88%, *P* = 0.573). The whole cost of VATS (overall hospital charges including the consumables) was significantly higher than those in the thoracotomy group (*P* < 0.001). The authors concluded that VATS is safe with satisfactory outcomes in selected patients with bronchiectasis.

Zhou *et al.* [4] retrospectively compared the outcomes of lobectomies using thoracotomy in 21 patients versus complete video-assisted thoracoscopic surgery (cVATS) in 35 patients. The selection of patients for both procedures was based on localization of bronchiectasis by HRCT, adequacy of the cardiopulmonary reserve, the significance of symptoms and failure of the medical treatment. Severe scarring or adhesions on CT scan was a contraindication for the cVATS. The authors did not give much information on comparing preoperative parameters except for age, gender and symptomatic history. The conversion rate to thoracotomy was 5.7% because of fused fissures, severe adhesions, or massive neovascularization within the hilum, with no conversion due to major bleeding. cVATS resulted in less blood loss (*P* = 0.015), a trend towards shorter operative time (*P* = 0.071) and more charges (*P* < 0.0001). The authors advocated that the high cost of VATS is the principal obstacle to its popularization. The median follow-up time was 89 months (range: 39–123 months) in the thoracotomy group vs 29.7 months (range: 11–53 months) in the cVATS group. There was no significant difference in postoperative complications and effectiveness (asymptomatic plus significant symptom improvement) between the 2 groups (*P *>* *0.05). In the cVATS group, the complications include postoperative air leak (*n* = 5) and atelectasis (*n* = 1). The authors concluded that cVATS lobectomy is safe and effective for patients with localized bronchiectasis.

Hao *et al.* [5] analysed 99 consecutive patients who underwent lung resection to treat bronchiectasis through VATS (*n* = 49) or open lung resection (*n* = 50). The indications for surgery were localized bronchiectasis documented by HRCT, obvious symptoms (chronic productive cough, repeated or significant haemoptysis and recurrent pulmonary infections) and failure of medical treatment. The selection of VATS or thoracotomy was based on the preference of surgeons. The patients in the VATS group were older than those in the open group (52.84 ± 8.66 vs 49.25 ± 10.74 years, *P* = 0.025). Severe pleural adhesion did not affect the decision for VATS as it was observed in 8 patients (16.3%). No conversion to thoracotomy occurred in the VATS group. The lung resection did not include lobectomy only, but it was carried out in most patients, followed by lobectomy, bilobectomy, segmentectomy and pneumonectomy. VATS resulted in shorter duration of chest tube placement (*P* = 0.01) and length of hospital stay (*P* = 0.04) but longer operative time (*P* = 0.01). In the VATS group, postoperative complications occurred in 16.3% vs 18% in the thoracotomy group (*P* = 0.671). The complications in the VATS group include persistent air leak for >7 days (*n* = 4), pneumonia (*n* = 2), atelectasis (*n* = 1) and atrial arrhythmia (*n* = 1). During a mean follow-up of 33.6 months (range: 5–65 months), the symptoms disappeared in most of the patients with no significant difference between the 2 groups (35 in the VATS group vs 38 in the thoracotomy group, *P* = 0.694). The authors concluded that lung resection through VATS for patients with localized bronchiectasis is safe and efficient with good recovery.

Furthermore, Ceylan *et al.* [6] reviewed and compared 20 patients who underwent VATS lung resection and 30 patients who underwent thoracotomy. All patients had a preoperative work-up including chest CT, pulmonary function tests, bronchoscopy, sputum culture and bronchial lavage. The preoperative poor respiratory functions indicated bronchodilator treatment and respiratory physiotherapy. The indications for surgery were recurrent lower respiratory tract infection resistant to medical treatment, chronic productive cough and chronic recurrent or significant haemoptysis. Increasing the experience in VATS over time leads to change the selection criteria for the surgical technique. VATS was initially carried out for young patients without an additional medical condition, but this preference was changed over time with increasing experience in VATS. The conversion rate of VATS to thoracotomy was 10% due to dense pleural adhesions. Left lower lobectomy was performed in most of the patients, but pneumonectomy was performed in 2 patients (1 in each group) because of a destroyed lung. There was no significant difference between the 2 groups in intraoperative complications (*P* = 0.54), mean operation time (*P* = 0.48), duration of chest tube placement (*P* = 0.25) and length of hospital stay (*P* = 0.25). The mean pain scores (measured by visual analog scale) were lower in the first 5 postoperative days after VATS. There was no significant difference in the complication rates between the 2 groups (38.9% vs 37.5%, *P* = 1). The prolonged air leakage was the most common postoperative complication in both groups (27.8% after VATS and 18.8% after thoracotomy). The authors concluded that VATS is safe, feasible and effective for patients with bronchiectasis, especially those with localized disease. The indications of VATS for more challenging cases can be expanded with increasing experience.

## CLINICAL BOTTOM LINE

We conclude that although there is limited high-level evidence, retrospective studies have suggested that VATS could significantly reduce complication rate, postoperative blood loss, pain scales, length of hospital stay and chest tube duration compared to open lung resection in selected patients with localized bronchiectasis.

**Conflict of interest:** none declared. 

### Reviewer information

Interactive CardioVascular and Thoracic Surgery thanks Ahmed Boseila and the other, anonymous reviewer(s) for their contribution to the peer review process of this article.
